# Genetic changes in the *FH* gene cause vagal paraganglioma

**DOI:** 10.3389/fendo.2024.1381093

**Published:** 2024-04-24

**Authors:** Anastasiya V. Snezhkina, Vladislav S. Pavlov, Dmitry V. Kalinin, Elena A. Pudova, George S. Krasnov, Asiya F. Ayupova, Anastasiya A. Kobelyatskaya, Alexey A. Dmitriev, Dmitrii A. Atiakshin, Maria S. Fedorova, Anna V. Kudryavtseva

**Affiliations:** ^1^ Engelhardt Institute of Molecular Biology, Russian Academy of Sciences, Moscow, Russia; ^2^ Vishnevsky Institute of Surgery, Ministry of Health of the Russian Federation, Moscow, Russia; ^3^ Scientific and Educational Resource Center “Innovative Technologies of Immunophenotyping, Digital Spatial Profiling and Ultrastructural Analysis”, RUDN University, Moscow, Russia

**Keywords:** head and neck paraganglioma, vagal paraganglioma, mutation profile, LOH, biallelic inactivation, *FH*

## Abstract

Vagal paraganglioma (VPGL) is a rare neuroendocrine tumor that originates from the paraganglion associated with the vagus nerve. VPGLs present challenges in terms of diagnostics and treatment. VPGL can occur as a hereditary tumor and, like other head and neck paragangliomas, is most frequently associated with mutations in the *SDHx* genes. However, data regarding the genetics of VPGL are limited. Herein, we report a rare case of a 41-year-old woman with VPGL carrying a germline variant in the *FH* gene. Using whole-exome sequencing, a variant, *FH* p.S249R, was identified; no variants were found in other PPGL susceptibility and candidate genes. Loss of heterozygosity analysis revealed the loss of the wild-type allele of the *FH* gene in the tumor. The pathogenic effect of the p.S249R variant on FH activity was confirmed by immunohistochemistry for S-(2-succino)cysteine (2SC). Potentially deleterious somatic variants were found in three genes, *SLC7A7*, *ZNF225*, and *MED23*. The latter two encode transcriptional regulators that can impact gene expression deregulation and are involved in tumor development and progression. Moreover, *FH*-mutated VPGL was characterized by a molecular phenotype different from *SDHx*-mutated PPGLs. In conclusion, the association of genetic changes in the *FH* gene with the development of VPGL was demonstrated. The germline variant *FH*: p.S249R and somatic deletion of the second allele can lead to biallelic gene damage that promotes tumor initiation. These results expand the clinical and mutation spectra of *FH*-related disorders and improve our understanding of the molecular genetic mechanisms underlying the pathogenesis of VPGL.

## 1 Introduction

Vagal paraganglioma (VPGL) is a neuroendocrine tumor with an extremely low annual incidence rate (1 per 100,000) (1). VPGL arises from a parasympathetic paraganglion within or adjacent to the vagus nerve accounting for approximately 13% of head and neck paragangliomas (HNPGLs) (2). The clinical signs and symptoms of VPGL vary; the most frequent symptoms include neck mass, pulsatile tinnitus, pharyngeal mass, hoarseness, and hearing loss. Metastasis occurs in 16–19% of cases (3). Up to 40% of VPGLs are characterized by multicentricity (multifocal cases) and predominantly occur together with HNPGLs in other locations (4). VPGL present challenges in terms of diagnostics and treatment. Despite the use of various instrumental diagnostic methods (computed tomography [CT], magnetic resonance imaging, and angiography), the diagnosis of VPGL is often made at the time of surgery. Tumor resection carries the risk of cranial nerve damage but remains the only treatment option for individuals diagnosed with VPGL.

VPGL, a part of PPGL, is highly heritable (5). However, the genetics of VPGL have been poorly investigated owing to its rarity. Data on VPGL are mostly limited to reports on cases and case series, usually devoted to the clinical description of tumors, treatment experience, and patient management. Recently, we presented a series of genetic analyses of HNPGLs covering VPGLs. Germline mutations have been observed in PPGL susceptibility genes, such as *SDHB*, *SDHD*, *NF1*, *FH*, and *IDH2*, as well as in candidate PPGL-associated genes, *ACLY*, *OGDH*, and *PDHA2* (6). Most *SDHx*-mutated VPGLs were multifocal tumors that manifest simultaneously as carotid paragangliomas. Somatic variants of *ACO1*, *PIK3CA*, and *TP53* have been identified (6). Ding et al. reported germline mutations in *SDHB* and *MAP3K13* in malignant VPGLs (7). A few other studies have also found germline variants in the *SDHB* and *SDHD* genes (8–11). Familial VPGLs are associated with earlier disease onset (mean age, 45 vs. 60 years) and a higher risk of multifocality (78% vs. 23%) than sporadic tumors (4, 12). Due to the rarity of VPGLs, their etiology remains unclear. However, several conditions such as familial inheritance, genetic alterations in susceptibility genes, sex (female predominance), young age, Carney’s triad, and possibly chronic hypoxia may increase the risk of VPGL development (12, 13).

This study presents an analysis of the clinical phenotype and molecular genetics of VPGL with germline missense variant in the *FH* gene, which have been previously revealed in a comprehensive mutation profile analysis of HNPGLs (6). FH is considered a susceptibility gene for PPGLs; however, the frequency of *FH*-related cases is low (~ 1%) (14). VPGLs are predominantly caused by mutations in *SDHx* genes. To date, there have been no reports of *FH*-mutated VPGLs in the literature. Therefore, this case can be regarded as extremely rare and interesting.

## 2 Materials and methods

### 2.1 Patient

The patient was admitted to the Vishnevsky Institute of Surgery, Ministry of Health of the Russian Federation. Informed consent was obtained from the patient for molecular genetic studies and use of the data for scientific purposes and publication. The study was approved by the ethics committee of the Vishnevsky Institute of Surgery (ethics committee approval no. 007/18, October 2, 2018) and was performed in accordance with the Declaration of Helsinki (1964).

### 2.2 Immunohistochemistry

Pathomorphological studies were performed by the chief pathologist at the Department of Pathology, of Vishnevsky Institute of Surgery. Immunohistochemistry (IHC) was done on 3–5 μm thick sections made from the formalin-fixed, paraffin-embedded (FFPE) blocks with tumor and lymph node tissues of the patient as previously described (15). Immunoreactions were performed using the following primary antibodies: Chromogranin A (DAK-A3, DAKO, USA), Synaptophysin (MRQ-40, Cell Marque, USA), CD56 (123C3, DAKO), S100 protein (polyclonal, DAKO), Pancytokeratin (AE1/AE3, Biocare, USA), Ki67 (MIB-1, DAKO), SDHB (21A11AE7, Abcam, UK), FH (monoclonal, clone J-13, from Santa Cruz Biotechnology, USA), 2SC (polyclonal, Cambridge Research Biochemicals, UK), and 5-hydroxymethylcytosine (5-hmC) (polyclonal, Active Motif, USA). Secondary antibodies were conjugated to horseradish peroxidase (HRP) and detected using a Histofine DAB-2V system (Nichirei Biosciences, Japan). Automated staining was performed using a Lab Vision Autostainer 360-2D (Thermo Fisher Scientific). The slides were visualized using an Axio Imager 2 microscope (Carl Zeiss Microscopy, Germany). Stromal cells were used as internal positive controls for FH and 5-hmC, and as internal negative controls for 2SC. FH staining was considered “negative” in the absence of FH expression in tumor cells compared to a positive internal control and “positive” in other cases. 2SC staining was defined as “positive” if tumor cells displayed strong/diffuse staining in cytoplasm, nuclear, or both compared to negative internal control and “negative” in other cases. Slides were classified as “low 5-hmC” or “high 5-hmC” if tumor cells showed absent/low staining or slightly inferior/equivalent staining compared to markedly high staining of endothelial cells, respectively. Sporadic VPGL (without mutations in any susceptibility genes) and normal lymph node tissues were used as external negative controls for 2SC IHC (**Supplementary Figure S1**). Negative reagent controls for primary antibodies and the detection system were used to ensure the specificity of the IHC tests. Uterine leiomyomatous tissue from patients with hereditary leiomyomatosis was used as an external positive control for 2SC staining, and as an external negative control for FH immunoreactivity (**Supplementary Figure S1**). This control was kindly provided by Alexandra Asaturova, head of the Pathology Department, Gynecology and Perinatology Named after Academician V.I. Kulakov of Ministry of Healthcare of Russian Federation.

### 2.3 Whole-exome sequencing

Whole-exome sequencing was performed on tumor and normal (lymph node) tissues of the patient. Genomic DNA was extracted using a High Pure FFPET DNA Isolation Kit (Roche, Basel, Switzerland). Exome libraries were prepared with a TruSeq Exome Library Prep Kit (Illumina, USA) and sequenced using an Illumina NextSeq 500 system under 76 × 2 bp paired-end mode. The raw reads were aligned to the reference human genome (GRCh37/hg19) using BWA-MEM (16). Default parameters were used to start the alignment. Secondary (supplementary) alignments in the BWA output were removed with samtools *[samtools view -F 2048]* (17). Mapping statistics were obtained with samtools flagstat. The BAM files were preprocessed using Picard-tools (18) *[picard.jar FixMateInformation ADD_MATE_CIGAR=true]*. Then, duplicated reads were identified and marked *[picard.jar MarkDuplicatesWithMateCigar MINIMUM_DISTANCE=600]*. Germline variant calling was performed using GATK4 HaplotypeCaller, including the options *-A StrandBiasBySample, -A StrandOddsRatio, -A BaseQualityRankSumTest, -A MappingQualityRankSumTest, -A RMSMappingQuality, -A ReadPosRankSumTest, and -A FisherStrand* (19). Other parameters were set to default, except for *–max-reads-per-alignment-start 0*. GATK VariantFiltration was used for germline variant filtering *[SNVs - QD < 2.0, QUAL < 35.0, MQ < 40, MQRankSum < -12.5, FS > 60.0, SOR > 3.0, ReadPosRankSum < -8.0; indels - QD < 2.0, QUAL < 33.0, FS > 200.0, ReadPosRankSum < -20.0]*. Somatic variants were discovered using GATK Mutect2 (20). First, we created a list of variants observed in normal tissues, which were obtained from patients with HNPGLs (PoN, panel of norms) (6), with the GATK Mutect2, GenomicsDBImport and CreateSomaticPanelOfNormals tools. Then we supplied it to the final GATK Mutect2 search for tumor-paired sample which was started with the default parameters except for *max-reads-per-alignment-start 0, –f1r2-tar-gz <filename>, –germline-resource <gnomad_vcf>, -A StrandBiasBySample, -A StrandOddsRatio, -A AS_StrandOddsRatio*. Somatic variant filtration was performed with GATK FilterMutectCalls. However, because FFPE samples were examined, preliminary steps were performed to eliminate FFPE artifacts and potential cross-sample contamination. Orientation bias artifacts were evaluated using GATK LearnReadOrientationModel. Read counts supporting reference, alternate, and other alleles for GnomAD known SNP sites were calculated using the GATK GetPileupSummaries tool (with the *–min-mapping-quality 27* argument). Cross-sample contamination and tumor segmentation were evaluated using GATK CalculateContamination. Finally, GATK FilterMutectCalls was run with the derived information on tumor segmentation, cross-sample contamination, and orientation bias. All variants were annotated using ANNOVAR (21). The identified variants were filtered based on objective criteria, including mutation region (exonic and splicing), mutation type (missense, nonsense, insertion, deletion, stop-loss, start-loss), population allele frequency (<0.01, gnomAD), genomic region conservation score (>0.5, phastCons), clinical significance (not benign, ClinVar), and predicted as deleterious by more than 3 pathogenicity prediction algorithms (SIFT, PolyPhen2, LRT, and others). InterVar (22) and Varsome (23) tools were used for clinical interpretation of genetic variants. To verify all variants of interest, the IGV browser was utilized (24). The mutational load (ML) was calculated using an algorithm previously developed for FFPE PPGL samples (25). Copy number variations were analyzed using the beta allele frequency (BAF) method (6).

A total of 34.4 and 93.9 million reads were identified in normal and tumor samples, respectively; of these, 97.8% and 96.52% were mapped. 91.5% and 87.9% of reads were properly paired; 1.63% and 2.27% of reads were singletons; 16.1% and 42.9% of reads were duplicates.

### 2.4 Transcriptome sequencing

Total RNA was isolated from FFPE tumor tissues using a High Pure FFPET RNA Isolation Kit (Roche) and subjected to cDNA library preparation with a TruSeq Stranded Total RNA Ribo-Zero H/M/R Gold Kit (Illumina) following the manufacturer’s protocols. Sample sequencing was performed along with the collection of 104 HNPGLs on an Illumina NextSeq 500 system in a single-end run with a sequencing read length of 76 bp. Totally, 51.3 million reads (read quality > 30) were obtained for the sample. Primary bioinformatics analysis of raw sequencing data included quality control using FastQC (26) and read trimming, filtering, and adapter removal using Trimmomatic (27). Further sequencing reads were aligned using STAR (28) with the GRCh38.p12. The parameters that differed from the default were: *outFilterMismatchNmax – 6, outFilterMultimapNmax – 1, quantMode – TranscriptomeSAM, outSAMstrandField – intronMotif, outFilterIntronMotifs – RemoveNoncanonicalUnannotated*. The mapped reads were counted at the transcriptional level using featureCounts (Subread package) (29). Gene Set Variation Analysis (GSVA) was performed using the GSVA 1.46.0 (30) and clusterProfiler 4.6.2 (31) packages from Bioconductor, using the KEGG database for *SDHx*-mutated and non-mutated tumors. A total of 340 KEGG pathways were identified. The pathways identified with a p-value < 0.05 were sorted based on the [(1-adj. p-value)*(1-p-value)*absolute LogFC] score to identify the most significant and striking changes. A hierarchical clustering analysis was performed using the top 50 biological pathways based on this score. The clinical and pathological characteristics of the patient cohort with HNPGLs are shown in **Supplementary Table S1**.

### 2.5 Loss of heterozygosity analysis

LOH analysis was performed in tumor and normal tissues of the patient using three dinucleotide microsatellite repeats flanking the *FH* locus (1q43, GRCh37/hg19): D1S304, D1S204, and D1S321 (1q43), D1S235 (1q42.3) and D1S423 (1q44), as well as seven microsatellite markers located at different regions on chromosome 11: D11S1984, D11S1313, D11S907, D11S4088, D11S969, D11S1339, and D11S5030) as described in (32). Polymerase chain reaction (PCR) was performed using primers for microsatellites described previously (33). The obtained fluorescence-labeled PCR products were processed on a NANOPHORE-05 (Syntol, Russia) and analyzed using GeneMarker software (SoftGenetics, USA). A score < 0.7 or > 1.3 indicates LOH.

## 3 Results

### 3.1 Case description

A 42-year-old woman was referred to the Vishnevsky Institute of Surgery, Ministry of Health of the Russian Federation, with a mass on the right side of the neck, which had been growing for a long time. The patient had experienced frequent episodes of throat pain since the age of 26 years. She underwent tonsillectomy, which had no positive effects. She also complained of hoarseness.

**Figure 1:**
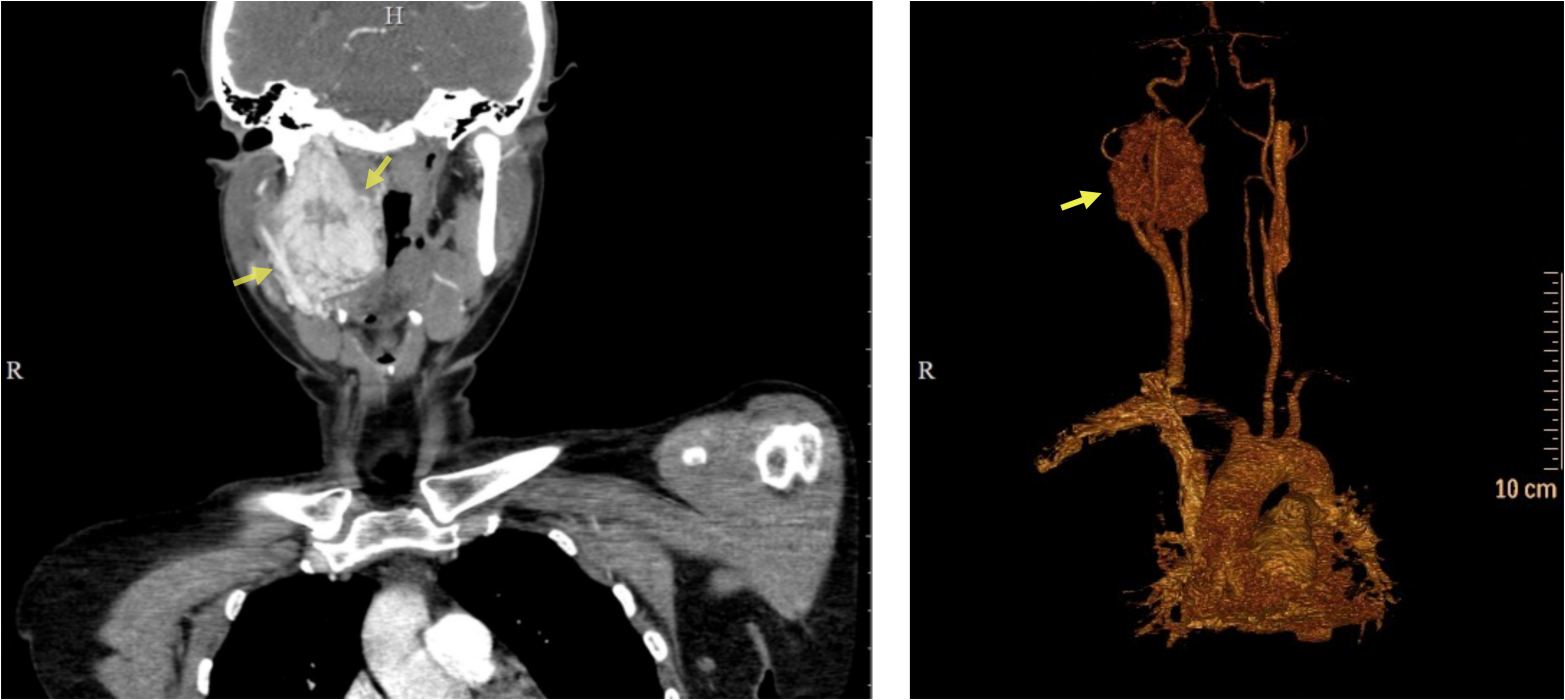
Preoperative computed tomography of the patient’s head and neck. CT scan (left), 3D reconstruction (right). Yellow arrows indicate the tumor.

A computed tomography (CT) revealed a solid tumor in the area of the right carotid artery bifurcation, with an oval shape and clear smooth contours, measuring 47 × 30 × 70 mm. An early arterial phase study revealed bright heterogeneous accumulation of the contrast agent and lobulations with hypodense central areas. Laterally, it deformed the surface of the neck and pushed the larynx. The lower pole of the lesion was located medial to the common carotid artery (CCA) bifurcation, in the area of the fork of the internal carotid artery (ICA) and external carotid artery (ECA), pushing the vessels anteriorly. Superiorly the lesion spread to the base of the skull (**Figure 1**).

In 2018, the patient underwent surgical tumor removal. During the procedure, the CCA, ICA, and ECA were isolated. The ICA was surrounded by the tumor up to its entry into the skull. When isolating the carotid arteries, three enlarged lymph nodes had to be removed. The tumor originated from the vagus nerve and partly from the sympathetic trunk. The vagus nerve was carefully dissected, ligated and sutured. During isolation of the ICA from the tumor, hypervascularization of the mass was noted due to active bleeding. The ICA was isolated up to the cranial entrance and requiring expansion of access, crossing of the digastric muscle, and removal of the styloid process. Following isolation of the ICA, the paraganglioma was excised from the base of the skull. It is noteworthy that the vagus nerve expanded to approximately 1 cm at the entrance to the skull. Careful hemostasis, wound drainage, and layer-by-layer suturing of the wound were performed after tumor extraction. The patient was transferred to the intensive care unit without experiencing any neurological symptoms.

**Figure 2:**
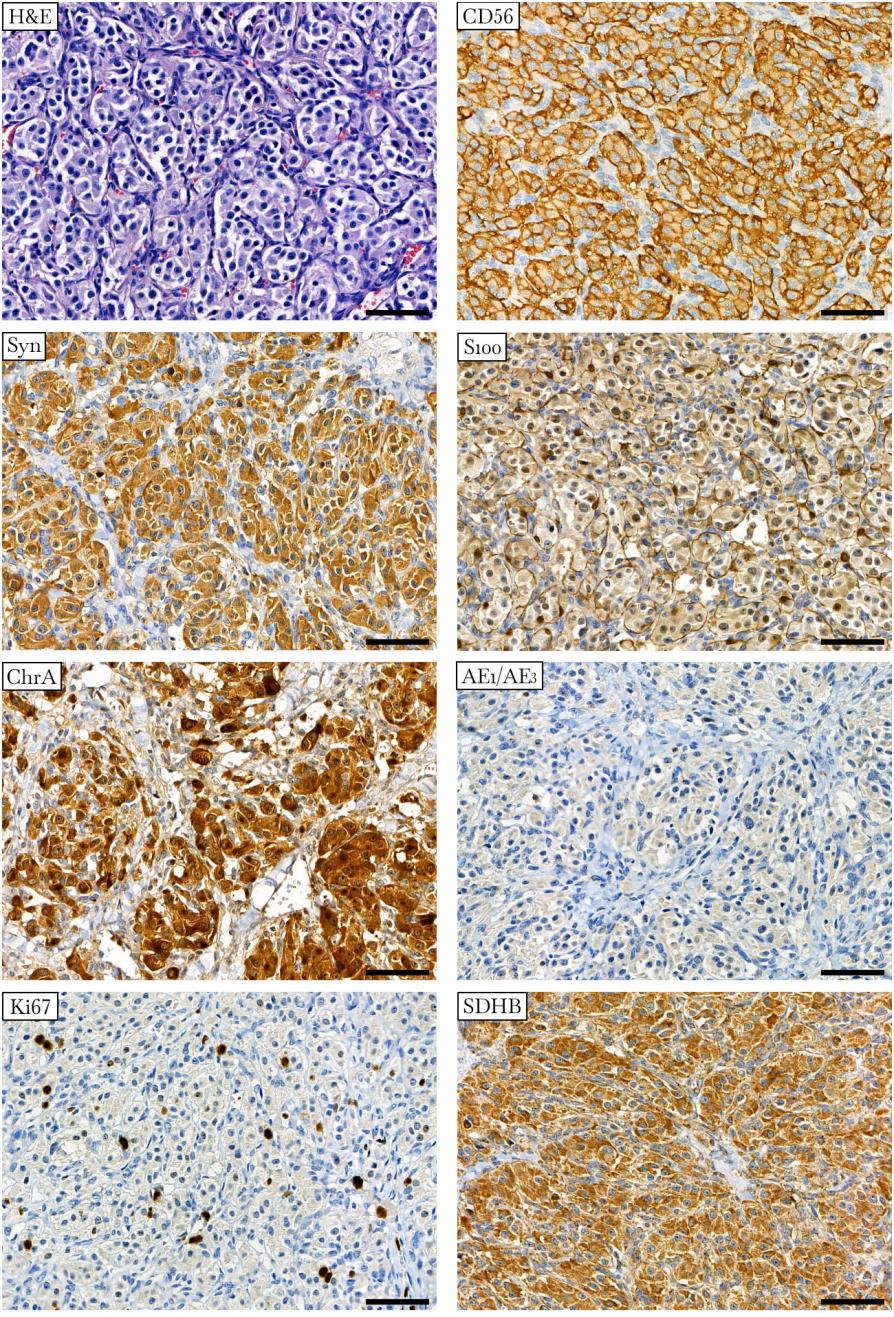
Histologic and immunohistochemical definition of paraganglioma. Hematoxylin-eosin (H&E) staining displays a specific “Zellballen” growth pattern with small nests of chief cells surrounded by supporting cells and further separated by fibrovascular stroma. Chief tumor cells stain positive for CD56, synaptophysin (Syn), chromogranin A (ChrA), and SDHB antibodies and negative for pancytokeratin (AE1/AE3). S100 staining occurs predominantly in sustentacular cells. Ki67 stains approximately 2% of the nuclei. ×400 magnification, scale bar 50 μm.

Histological examination of the resected tumor confirmed paraganglioma (**Figure 2**). Hematoxylin and eosin staining revealed a Zellballen structure typical of PPGLs. The chief tumor cells were positive for chromogranin A, synaptophysin, and CD56 antibodies, indicating a neuroendocrine tumor. S100 protein was expressed in sustentacular cells. The tumor cells tested negative for pancytokeratin. Ki67 stained about 2% of the cells. SDHB was positively expressed in tumor cells, suggesting no deleterious mutation in the *SDHx* genes and the presence of a stable succinate dehydrogenase complex (15, 34).

### 3.2 Mutation profiling and biological pathways enrichment

To reveal tumor-associated genetic changes, whole-exome sequencing was performed on the tumor and normal (lymph node) tissues of the patient as described previously (6). FFPE lymph node tissue slides containing normal cells were examined by a pathologist. Based on these results, a heterozygous germline variant, NM_000143: c.747T>A, p.S249R (chr1:241669460), was identified in the *FH* gene, with a variant allele frequency (VAF) of 0.57 in tumor and 0.45 in lymph node. This variant was recently submitted to the ClinVar database and was classified as a variant of uncertain significance. The *FH*: p.S249R variant was characterized by low population frequency (close to 0), high genomic region conservation score (0.89, set of 20 placental mammals), and “strong deleterious” predicted by all used *in silico* prediction algorithms (**Table 1**). According to the ACMG/AMP 2015 guideline (InterVar), this variant was interpreted as “uncertain significance” in the same way as in the GeneBe platform, whereas Varsome classified this variant as “likely pathogenic.” In the protein crystal structure of FH (35), serine 249 is located in the alpha chain in the central domain and forms a hydrogen bond with asparagine 310, another monomer in the functional homotetrameric enzyme (**Supplementary Figure S2**) (35–37). The substitution of serine 249 for arginine can lead to the destabilization of the tetrameric assembly and dynamics, leading to reduced FH activity. No mutations were found in other PPGL susceptibility genes (*EGLN1*, *EGLN2*, *MDH2*, *SDHA*, *SDHB*, *SDHC*, *SDHD*, *SDHAF2*, *MAX*, *RET*, *TMEM127*, *VHL*, *EPAS1*, *NF1*, *H3F3A*, *IDH1*, *IDH2*, *ATRX*, and *HRAS*) and 60 candidate genes in the reported panel (38). The identified *FH*: p.S249R variant was visualized using IGV (24) and was verified using Sanger sequencing (**Supplementary Figure S3**).

**Table 1:** Germline and somatic variants identified in the patient.

Gene	Variant description	Varianttype	Variant status	VAF	Average coverage	Population allele frequency (gnomAD)	Pathogenicity prediction algorithms	InterVar	Varsome
*FH*	NM_000143: c.747T>A, p.S249R (chr1:241669460)	Missense	Germline	0.57 (tumor), 0.45 (lymph node)	183	0	Polyphen2 – D, SIFT – D, LRT – D, MutationTaster – D, CADD – 1, DANN - 1	US	LP
*SLC7A7*	NM_001126105: c.1266delT, p.I422fs (chr14: 23243304)	Frameshift deletion	Somatic	0.13	130	0	NA	NA	LP
	NM_001126105: c.1246_1262del, p.L416fs (chr14: 23243308)		Somatic	0.06	127	0	NA	NA	LP
*ZNF225*	NM_013362: c.1777_1860del, p.593_620del (chr19: 44636543)	In-frame deletion	Somatic	0.11	159	0	NA	NA	LP
*MED23*	NM_004830: c.2317G>A, p.E773K (chr6: 131919805)	Missense	Somatic	0.09	124	0.00003	Polyphen2 – D, SIFT – D, LRT – D, MutationTaster – D, CADD – 1, DANN - 1	US	US

VAF, variant allele frequency; D, deleterious; T, tolerated; CADD and DANN scores ranged from 0 to 1; a larger number indicates a higher probability to be damaging. NA, data unavailable; US, uncertain significance; LP, likely pathogenic.

A total of 21 somatic variants that affected 18 genes were identified. Potential deleterious somatic variants were found in only three genes (*SLC7A7*, *ZNF225*, and *MED23*) with variant allele frequency (VAF) ranging from 0.06 to 0.13 (**Table 1**). The *SLC7A7* gene carried two frameshift deletions. Mutational load (ML) was calculated based on exome sequencing data. VPGL was characterized by a low ML, 0.01 at VAF 0.15 (or 0.06 at VAF 0.2). An analysis of copy number variations was performed for all chromosomes using the BAF method. The tumor showed a potential loss of chromosomes 1, 11, and 20 in some cells (**Supplementary Figure S4**).

RNA sequencing was performed on the tumor tissue of the patient, along with the collection of 104 HNPGLs. Only the mutational profile has been previously studied for a subset of these tumors (6). Hierarchical clustering analysis of the top-50 enriched KEGG biological pathways between *SDHx*-mutated and non-mutated tumors (GSVA, P < 0.05) was conducted. This revealed that *FH*-mutated VPGL were related to a cluster consisting predominantly of non-*SDHx*-mutated HNPGLs. Genes upregulated in *SDHx*-mutated tumors were mainly downregulated in *FH*-mutated VPGL. Sample clustering did not depend on carotid or vagal tumor localization (**Supplementary Figure S5**).

### 3.3 Biallelic inactivation of the *FH* gene

Based on BAF analysis (6), probable *FH* wild-type allele loss was observed. To validate these data, LOH analysis was performed on the tumor and normal tissues of the patient using three dinucleotide microsatellite repeats located near the *FH* locus (1q43, GRCh37/hg19), D1S304, D1S204, and D1S321 (1q43), as well as two more distant markers, D1S235 (1q42.3) and D1S423 (1q44) (**Supplementary Figure S6**). Two microsatellite markers, one close to the *FH* gene (D1S321) and the left distant marker (D1S235) demonstrated LOH with allelic imbalance ratios of < 0.7 or > 1.3 (LOH = 1.36 and 1.41, respectively); the right distant marker (D1S423) showed retention of heterozygosity. The other microsatellite repeats, D1S304 and D1S204, were homozygous (noninformative). Microsatellite analysis confirmed allelic loss in regions near the *FH* locus, indicating inactivation of the normal copy of the gene (**Supplementary Figure S6**).

BAF analysis revealed a potential loss of chromosome 11 in *FH*-mutated tumors. The loss of chromosome 11 has previously been shown in PPGLs with germline mutations in *SDHAF2*, *SDHD*, *VHL*, and *SDHB* genes (39). To examine this result, LOH was analyzed for seven microsatellite markers located in different regions of chromosome 11 (D11S1984, D11S1313, D11S907, D11S4088, D11S969, D11S1339, and D11S5030) (**Supplementary Figure S6**). Three of these seven markers (D11S5030, D11S1984, and D11S1339) were not informative. Two microsatellite repeats showed LOH at 11p13 (D11S907) and 11q25 (D11S969) regions, whereas loci 11q.12.1 (D11S1313) and 11p15.5 (D11S4088) were characterized by the retention of both alleles in tumor tissue (**Supplementary Figure S6**). Thus, the summarized results from the BAF analysis and microsatellite examination indicated deletions of particular regions on chromosome 11 rather than the loss of the whole chromosome in the studied patient.

### 3.4 Fumarate hydratase deficiency

**Figure 3:**
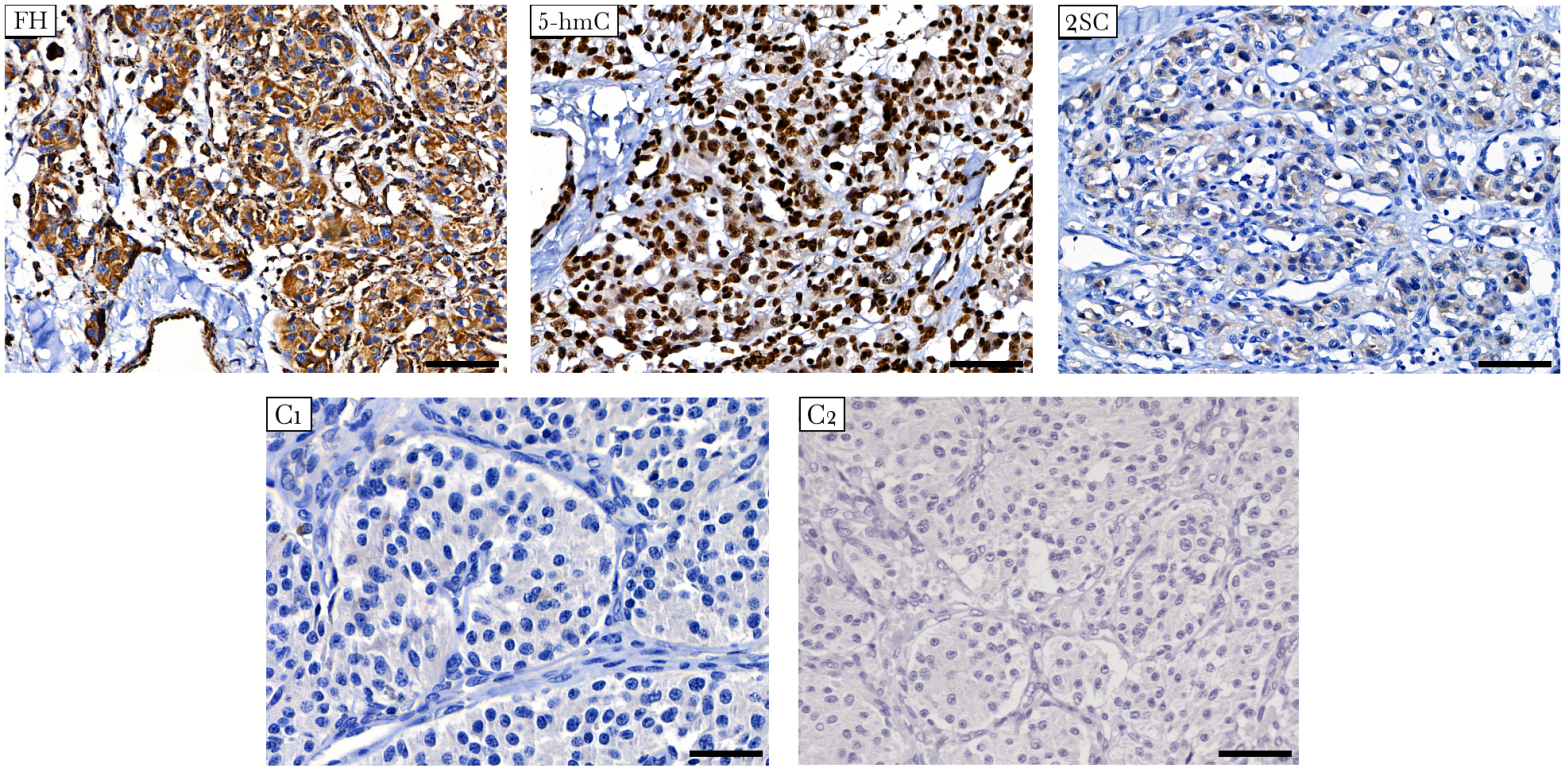
Immunostaining for FH, 5-hmC, and 2SC in *FH*-mutated VPGL. The tumor shows strong positive staining for FH (top left), diffuse cytoplasmic staining for 2SC (top right), and high nuclear labeling for 5-hmC (top middle). (C1-C2) Negative control reactions were performed for the antibodies used in the study. Negative immunostaining of 2SC in non-*FH*-mutated vagal paraganglioma (C1, bottom left). Isotype control image for 5-hmC and 2SC antibodies (C2, bottom right). Other control images can be found in **Supplementary Figure S1**. ×400 magnification, scale bar 50 μm.

The presence of FH deficiency in the tumor was estimated by IHC staining of two markers: FH and 2SC. The latter is a marker of loss or reduction in FH enzymatic activity associated with elevated fumarate levels, resulting in increased protein succination and production of 2SC (40). Strong positive staining for FH was observed in the studied tumor, indicating that the mutant protein was stable and detectable using the anti-FH antibody (**Figure 3**). Simultaneously, the tumor showed positive diffuse cytoplasmic staining for 2SC, a marker of deficient FH activity (41).

In addition, the impact of the *FH* variant on methylation was studied using 5-hmC IHC. Accumulation of succinate and fumarate blocks the hydroxylation of 5-mC to 5-hmC catalyzed by ten-eleven translocation (TET) enzymes, leading to the loss of 5-hmC, which is an evidence of DNA hypermethylation (42). The 5-hmC level was assessed by comparison with that of adjacent endothelial cells as a positive internal control. In tumor cells, 5-hmC staining was characterized by nuclear labeling with an intensity equivalent to that in endothelial cells (**Figure 3**).

## 4 Discussion

The *FH* gene encodes for the fumarate hydratase enzyme, which is a component of the tricarboxylic acid (TCA) cycle and catalyzes the conversion of fumarate to malate. *FH* is a housekeeping and tumor suppressor gene, and its germline inactivating mutations cause severe pathologies such as uterine and cutaneous leiomyomata and renal cell carcinoma (43). Recently, several studies revealed *FH* as a new susceptibility gene for PPGLs, with a mutation frequency varying from 0.83% to 2.8% (**Table 2**) (14, 44–46). We detected an *FH* mutation frequency in HNPGLs of 0.66%, which is lower than that reported for PPGLs predominantly enriched with pheochromocytomas (PCCs). Only four cases of *FH*-mutated HNPGLs (three carotid body tumors and one case of VPGL - the index case) have been reported to date (14, 45, 47). The age of the identified PPGL patients with germline *FH* variants ranged from 6–70 years, and somatic *FH* variants were found in patients aged 27–77 years. Importantly, *FH* mutations were associated with metastasis and multifocal development of PPGLs in approximately one-fourth of the cases (**Table 2**). The index patient with the germline *FH*: p.S249R variant was diagnosed with VPGL at 41 years of age; however, symptoms of the disease had been a concern for her for more than 16 years. No regional or distant metastases were detected at the time of diagnosis by CT or histopathological analyses. However, the patient lacked long-term follow-up; therefore, possible metastasis cannot be excluded. Thus, *FH*-related PPGLs appear to manifest at a young age and are likely to predispose to malignant tumors. Therefore, the identification of germline *FH* mutations and subsequent monitoring of carriers are important for early diagnosis and prevention of disease progression.

**Table 2:** Characteristics of reported PPGL cases with *FH* variants.

Patient	Sex	Age	Tumor	Metastasis	Multiple PPGL	Variant type	Variant status	Variant description	LOH	FH deficiency	*FH* variant frequency in the studied cohort (Germline)	Reference
1	F	63	PCC	No	Yes	Missense	Germline	c.349G>C, p.Ala117Pro	Yes	Yes	0.83%	(14, 47)
						Missense	Somatic	c.1043G > C, p.Gly348Ala				
2	F	20	PCC, extra-adrenal PGL	Yes	No	Splicing	Germline	c.268-2A>G, p.?	Yes	Yes		
3	M	28	Extra-adrenal PGL	Yes	Yes	Missense	Germline	c.1142C>T, p.Thr381Ile	Yes	Yes		
4	F	54	PCC, extra-adrenal PGL	Yes	Yes	Missense	Germline	c.580G>A, p.Ala194Thr	NA	NA		
5	M	70	HNPGL (carotid body)	No	No	Missense	Germline	c.986A>G, p.Asn329Ser	NA	NA		
6	M	6	PCC	No	No	Missense	Germline	c.1301G>A, p.Cys434Tyr	NA	NA	2.8%	(44)
7	M	41	PCC	No	No	Missense	Germline	c.157G>A, p.Glu53Lys	NA	NA		
8	NA	20	NA	NA	NA	NA	NA	NA	NA	NA	1%	(48)
9	NA	63	NA	NA	NA	NA	NA	NA	NA	NA		
10	M	30	PCC	No	Yes	Missense	Germline	c.1142C>T, p.Thr381Ile	NA	Yes	1.1%	(45)
11	F	36	Extra-adrenal PGL	No	No	Missense	Somatic	c.1516A>G, p.Met506Va	Yes	Yes		
12	F	69	PCC	No	No	Missense	Germline	c.222A>T, p.Arg74Ser	No	Yes		
13	M	77	Extra-adrenal PGL	No	Other tumor	Missense	Somatic	c.203A>G, p.Tyr68Cys	No	Yes		
14	F	59	PCC	No	Other tumor	Missense	Germline	c.1142C>T, p.Thr381Ile	NA	Yes		
15	F	32	PCC	No	Other tumor	Missense	Germline	c.1142C>T, p.Thr381Ile	NA	Yes		
16	M	57	HNPGL (carotid body)	Yes	No	Missense	Germline	c.700AC>T, p.Thr234Ala	Yes	Yes		
17	F	53	Extra-adrenal PGL	Yes	No	Missense	Germline	c.222A>T, p.Arg74Ser	NA	Yes		
18	F	33	Extra-adrenal PGL	No	No	Missense	Germline	c.817G>A, p.Ala273Thr	NA	NA	NA	(49)
19	F	36	Extra-adrenal PGL	No	No	Missense	Germline	c.817G>A, p.Ala273Thr	Yes	Yes	1.25%	(50)
20	F	27	PCC	No	Other tumor	Missense	Somatic	c.206G>Ab, p.Gly69Asp	No	Yes		
21	M	57	PCC, extra-adrenal PGL	No	Yes	Missense	Germline	c.193G>A, p.Asp65Asn	Yes	No		
22	F	46	Extra-adrenal PGL	No	No	Missense	Germline	c.799C>G, p.Pro267Ala	No	No		
23	M	50	PCC	No	No	Missense	Germline	c.1373C>T, p.Ala458Val	NA	NA		
24	M	30	PCC	No	Yes	Missense	Germline	c.1142C>T, p.Thr381Ile	NA	Yes	<1.1%	(45)
25	F	69	PCC	No	NA	Missense	Germline	c.222A>T, p.Arg74Ser	No	Yes		
26	F	59	PCC	No	Other tumor	Missense	Germline	c.1142C>T, p.Thr381Ile	NA	Yes		
27	F	32	PCC	No	Other tumor	Missense	Germline	c.1142C>T, p.Thr381Ile	NA	Yes		
28	M	57	HNPGL (carotid body)	Yes	NA	Missense	Germline	c.700A>G, p.Thr234Ala	Yes	Yes		
29	F	53	Extra-adrenal PGL	Yes	NA	Missense	Germline	c.222A>T, p.Arg74Ser	NA	Yes		
30	F	36	Extra-adrenal PGL	No	NA	Missense	Somatic	c.1516A>G, p.Met506Val	Yes	Yes		
31	F	41	HNPGL (vagal)	No	No	Missense	Germline	c.747T>A, p.S249R	Yes	Yes	0.66% (frequency only in HNPGLs)	Present study

F, female; M, male; NA, data unavailable.

The identified *FH* variant p.S249R was previously poorly described in the clinical databases. Its effect on protein was interpreted as “likely pathogenic” (Varsome) and “uncertain significance” (ACMG/AMP 2015). According to the protein crystal structure, the replaced amino acid (serine 249) is located in the central domain and is involved in the interactions of monomers in homotetrameric mitochondrial FH proteins. *FH* acts as a tumor suppressor, and the biallelic loss of this gene can cause cancer (51). LOH analysis revealed the loss of the wild-type *FH* allele in the studied tumor. Moreover, the potential loss of chromosome 11 predicted by BAF analysis was examined, but only specific chromosomal deletions were confirmed. Similar deletions are typical along chromosomes 1 and 20, which have also been detected with potential loss using BAF analysis.

The FH damage was confirmed by IHC staining of the 2SC. Tumor cells displayed 2SC positivity with simultaneous positive immunostaining for the FH protein. Immunostaining for FH and 2SC is widely used to detect FH deficiency caused by germline FH mutations in hereditary leiomyomatosis and renal cell carcinoma syndrome (52). A recent study demonstrated the rational utility of this approach for identifying *FH* variants in PPGL (45). Fuchs et al. screened 589 PPGLs using FH and 2SC staining and identified eight FH-deficient tumors, the majority of which had germline *FH* mutations. They also observed that FH-deficient tumors did not exhibit SDH complex deficiencies. This is consistent with our results, as *FH*-mutated VPGL were shown to have a positive SDHB expression pattern, indicating a stable SDH complex. The study of Fuchs et al. also demonstrated the high specificity of 2SC IHC for detecting FH deficiency in PPGLs associated with germline *FH* mutations. Interestingly, they found only one case of *FH*-mutated HNPGLs, a carotid body tumor. Similar to our case, the tumor showed positive FH and 2SC staining and carried a pathogenic/likely pathogenic *FH*: p.Thr234Ala variant. Buelow et al. reported several cases of positive FH and 2SC IHC results associated with pathogenic *FH*: p.R233H mutation in multiple cutaneous leiomyomas (53). Thus, our finding of positive FH expression and positive diffuse cytoplasmic staining in 2SC is consistent with the presence of a pathogenic/likely pathogenic missense mutation. Together with the results of LOH analysis, a positive FH pattern indicated the expression of a stable mutant protein. Simultaneously, the *FH*: p.S249R variant impaired FH activity and caused increased levels of protein succination, as reflected by positive diffuse 2SC IHC. This demonstrates the potential pathogenic effect of the *FH* p.S249R variant and the presence of FH deficiency in the tumor. We believe that the results obtained may provide evidence to classify the variant as “likely pathogenic” based on the following ACMG/AMP 2015 criteria: PS3, PM2, PP2 and PP3.

A deficiency of SDH or FH in successive reactions in the TCA cycle leads to the accumulation of the oncometabolites succinate and fumarate. Both succinate and fumarate can act as antagonists of α-ketoglutarate (α-KG) inhibiting α-KG-dependent dioxygenases, including JmjC domain-containing histone demethylases (KDMs) and the TET family of DNA hydroxylases that lead to alterations in histone and DNA methylation (42). FH- and SDH-deficient PPGLs are likely characterized by similar molecular phenotypes, which were confirmed by the common development of a hypermethylation phenotype and related gene expression profiles (47). Interestingly, the opposite results were observed. Cluster analysis of the biological pathways revealed that *FH*-mutated tumors did not display an “SDH-like” phenotype. This might be explained by the specificity of the molecular profiles of HNPGLs, which are not characterized by clear *SDHx*-mutated and non-mutated transcriptomic clusters compared with PCCs. However, an additional IHC test for a specific epigenetic marker, 5-hmC, showed the absence of DNA hypermethylation in the tumor and confirmed a different molecular phenotype of the studied *FH*-mutated tumor. Different DNA methylation has previously been shown in FH-deficient renal cell carcinoma (RCC) (54). Approximately 20% of FH-deficient RCC did not exhibited the CpG island methylator phenotype (CIMP), which was associated with lower incidence of metastasis. Thus, our result is consistent with the heterogeneity of DNA methylation in FH-deficient tumors and may also be related to benign tumor behavior. Notably, the clustering analysis of *FH*-related tumors was limited to one sample. Moreover, the sampling and assay designs were different from those in a previously reported study, which can also explain the specific sample clustering.

Somatic mutation profile analysis revealed three genes, *SLC7A7*, *ZNF225*, and *MED23*, with potentially deleterious variants. The *SLC7A7* gene encodes for the light subunit of a cationic amino acid transmembrane transporter that is involved in the activity of the y^+^L amino acid transport system (55, 56). No variants in *SLC7A7* have been previously reported in PPGLs; the role of short *SLC7A7* deletions in *FH*-mutated VPGL remains unclear. *ZNF225* and *MED23* encode proteins involved in the transcriptional activation of genes. ZNF225 is predicted to be a DNA-binding transcriptional activator specific for RNA polymerase II; however, little is known about this. MED23 is a subunit of the mediator complex that plays a key role in regulating gene expression via RNA polymerase II and other post-transcriptional steps (57). Interestingly, decreased *MED23* expression was correlated with malignant PCCs with a high degree of sensitivity but suboptimal specificity (58). Thus, somatic genetic alterations in transcriptional activators may be involved in the development of *FH*-mutated VPGL. These mutations could lead to the downregulation of many genes, as observed in the pathway enrichment analysis of the tumors (**Figure 3**).

## 5 Conclusion

The fourth case of *FH*-mutated HNPGLs has been reported; notably, this is the first reported case of VPGL. In this patient, the germline *FH* variant p.S249R co-occurred with LOH at the gene locus and FH deficiency. This strongly suggests that *FH* acts as a tumor suppressor gene in paragangliomas and may serve as evidence of variant pathogenicity. Despite the clear common change in cell metabolism driven by *FH* and *SDH* mutations, *FH*-mutated VPGL did not display an “SDH-like” molecular phenotype, indicating a different mechanism of tumor development. The identified somatic variants in the transcriptional activators ZNF225 and MED23 seem to be associated with the deregulation of gene expression and play a role in tumor pathogenesis and potential progression (particularly MED23). This case highlights the association between HNPGLs and hereditary *FH* mutations and firmly supports its consideration as a tumor susceptibility gene. Despite the rarity of *FH* mutations in PPGLs, carriers seem to be inclined toward early onset tumors with potential for metastasis. Genetic testing for germline mutations in *FH* is recommended for patients with HNPGL without variants in the *SDHx* genes, with subsequent screening of family members.

## Data availability statement

The datasets presented in this study can be found in online repositories. The names of the repository/repositories and accession number(s) can be found below: https://www.ncbi.nlm.nih.gov/, PRJNA993587.

## Ethics statement

The studies involving humans were approved by The ethics committee of the Vishnevsky Institute of Surgery, Ministry of Health of the Russian Federation. The studies were conducted in accordance with the local legislation and institutional requirements. The participants provided their written informed consent to participate in this study. Written informed consent was obtained from the individual(s) for the publication of any potentially identifiable images or data included in this article.

## Author contributions

AS: Conceptualization, Data curation, Funding acquisition, Investigation, Project administration, Supervision, Writing – original draft, Writing – review & editing. VP: Data curation, Formal analysis, Methodology, Visualization, Writing – review & editing, Writing – original draft. DK: Formal analysis, Methodology, Resources, Writing – original draft. EP: Methodology, Writing – original draft. GK: Methodology, Writing – original draft. AA: Formal analysis, Methodology, Writing – original draft. AK: Methodology, Writing – original draft. AD: Investigation, Writing – review & editing. DA: Formal analysis, Methodology, Writing – review & editing. MF: Investigation, Methodology, Writing – original draft. AK: Funding acquisition, Project administration, Writing – original draft, Writing – review & editing.
