# Supplementary material for: Genetic changes in the FH gene cause vagal paraganglioma
Source: Front Endocrinol (Lausanne). 2024 Apr 24;15:1381093. doi: 10.3389/fendo.2024.1381093 (PMC11076847; doi:10.3389/fendo.2024.1381093)
Supplement: Supplementary file 1 [file DataSheet_1.docx]

Table S1. Clinical and pathologic characteristics of patients with HNPGLs (subjected to transcriptome sequencing).

| **Characteristic** | **Number of patients, n** |
| --- | --- |
| Total patients | 98 |
| Total number of tumors | 104 |
| **Sex** | |
| Male | 26 |
| Female | 72 |
| **Age at diagnosis** | |
| ≥40 | 66 |
| <40 | 32 |
| Mean | 47.7 |
| **Tumor localization** | |
| Carotid paragangliomas | 76 |
| Vagal paragangliomas | 28 |
| **Tumor characteristics** | |
| Single | 94 |
| Bilateral/multiple | 7 |
| Recurrent | 6 |
| Metastasis | 1 |
| **Mutation** | |
| *SDHA* | 1 |
| *SDHB* | 13 |
| *SDHC* | 6 |
| *SDHD* | 22 |
| *FH* | 1 |


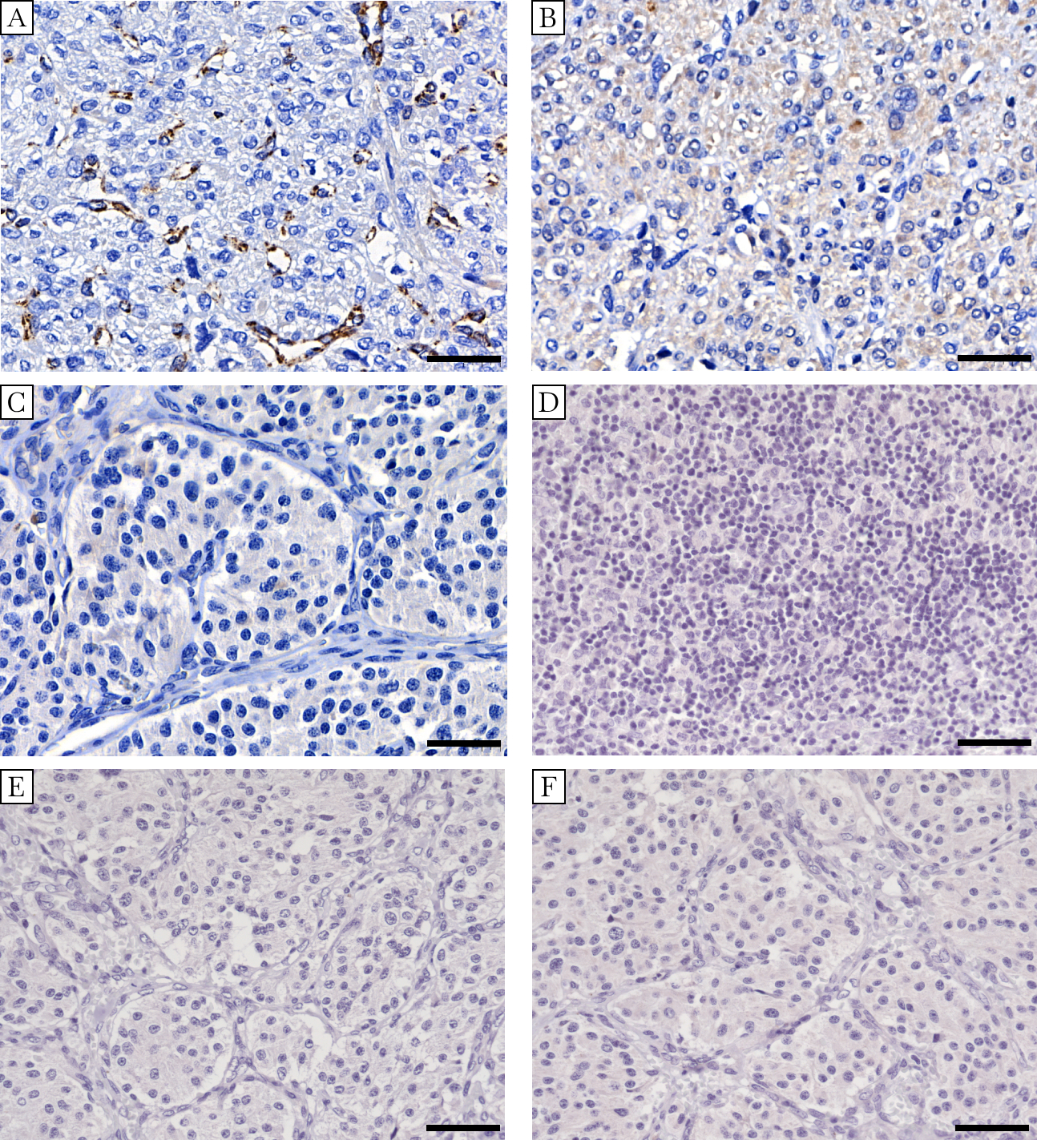


Figure S1. Immunohistochemical controls. (A) Negative immunostaining of FH in uterine leiomyomatous (external negative control). (B) Positive immunostaining of 2SC in *FH*-deficient uterine leiomyomatous (external positive control). (C) Negative immunostaining of 2SC in non-*FH*-mutated vagal paraganglioma (external negative control). (D) No primary antibody control image. (E and F) Isotype control images done with non-immune antibodies of the same isotype as those used in the study, rabbit IgG and mouse IgG, respectively. ×400 magnification, scale bar 50 μm.


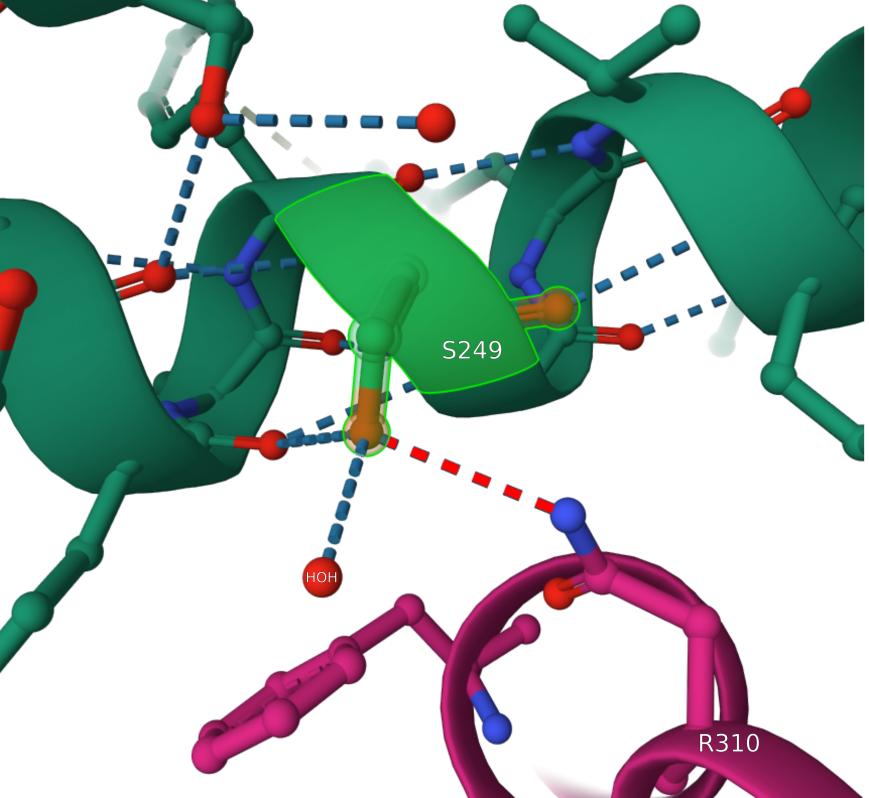


Figure S2. Surroundings of the SER249 site in the crystal structure of human fumarate hydratase (PDB ID: 3E04). Chain A and chain D of the tetramer are shown in green and pink, respectively. Amino acids are represented as balls and sticks. Dotted lines represent hydrogen bonds (hydrogen atoms are not shown). The dotted red line is the hydrogen bond between SER249 and ASN310, which is likely disrupted by the mutation in this case.


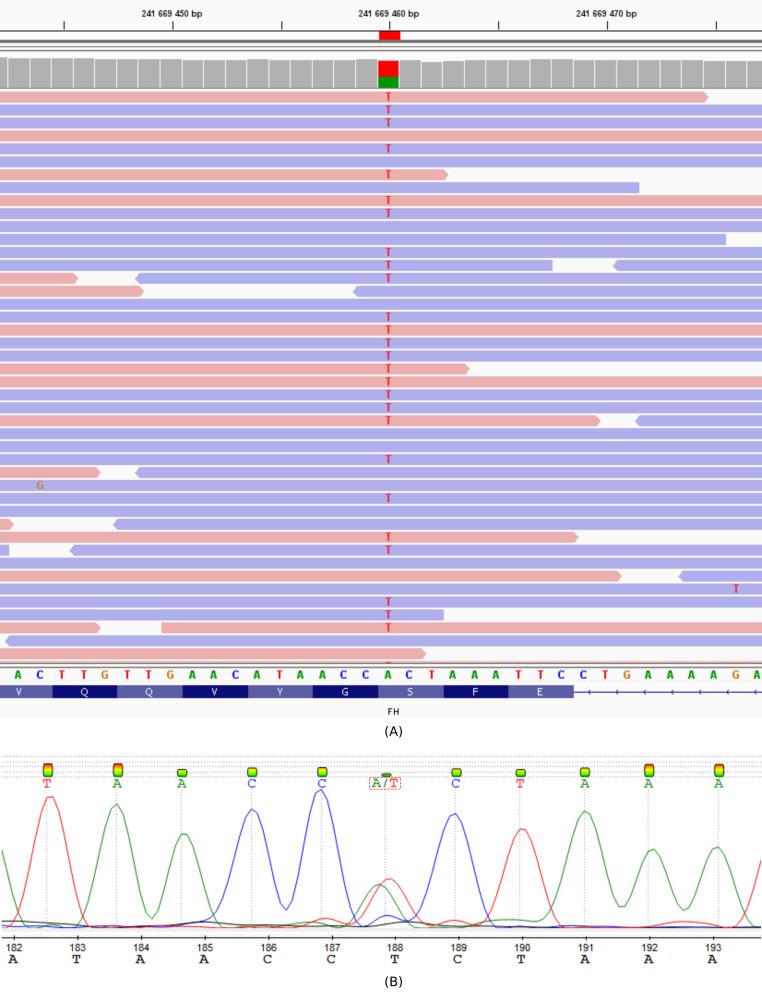


Figure S3. Validation of the *FH*: NM_000143: c.747T>A mutation with Sanger sequencing. (A) Exome sequencing data (variant nucleotides are marked by red color; forward and reverse reads are presented as pink and blue horizontal lines, respectively) IGV was used for visualization (https://www.igv.org/). (B) Sanger sequencing chromatogram.


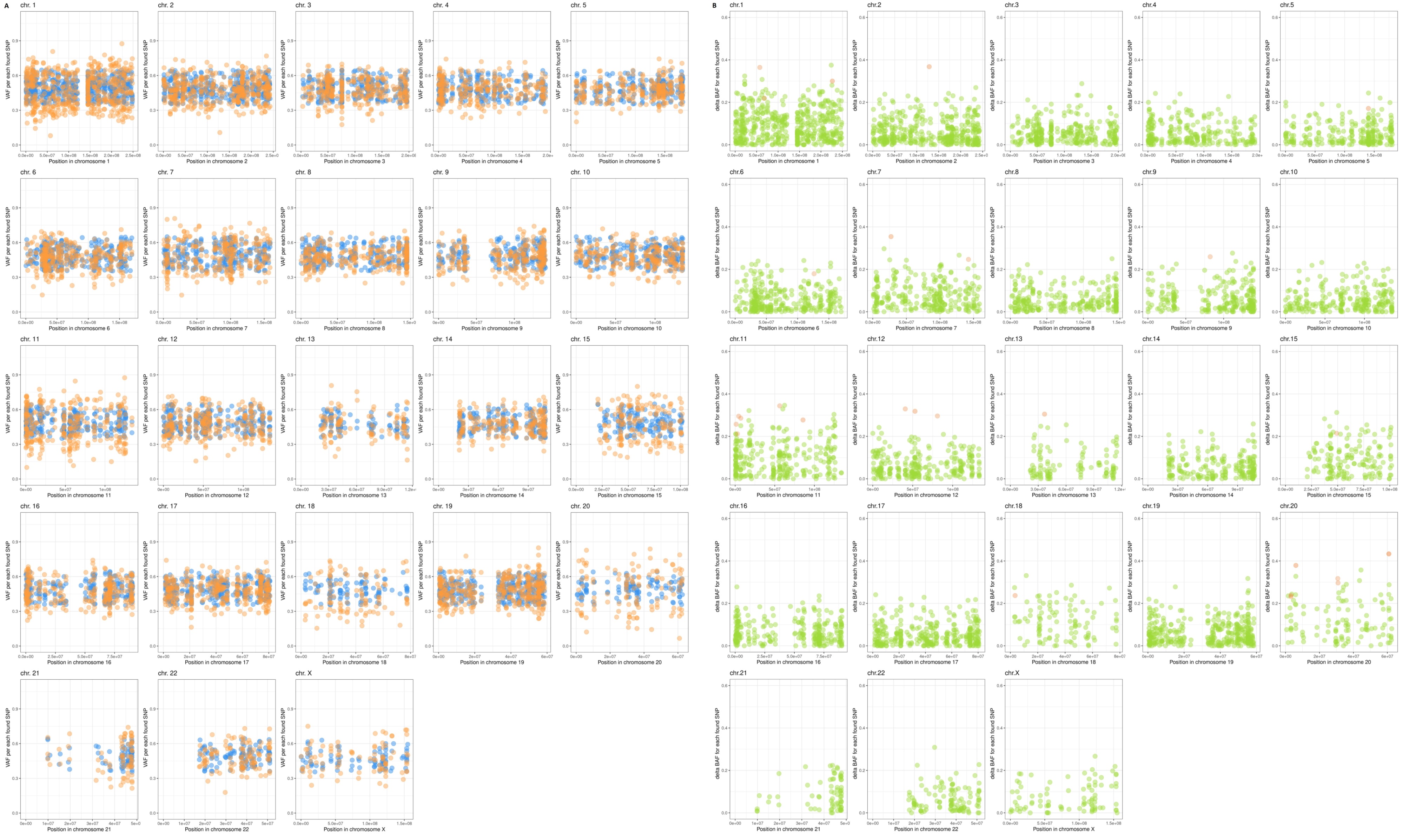


Figure S4. B-allele frequency (BAF) analysis of the tumor and lymph node of the patient with *FH*-mutated vagal paragangliomas. (A) Variant allele frequency (VAF) across all chromosomes of the tumor (orange dots) and lymph node (blue dots). (B) Delta-VAF across all chromosomes. Dots indicate SNPs with VAF that differed between the tumor and lymph node. They are colored orange if the delta-VAF value is greater than |0.15| at p ≤ 0.05, or green if it condition are not met.


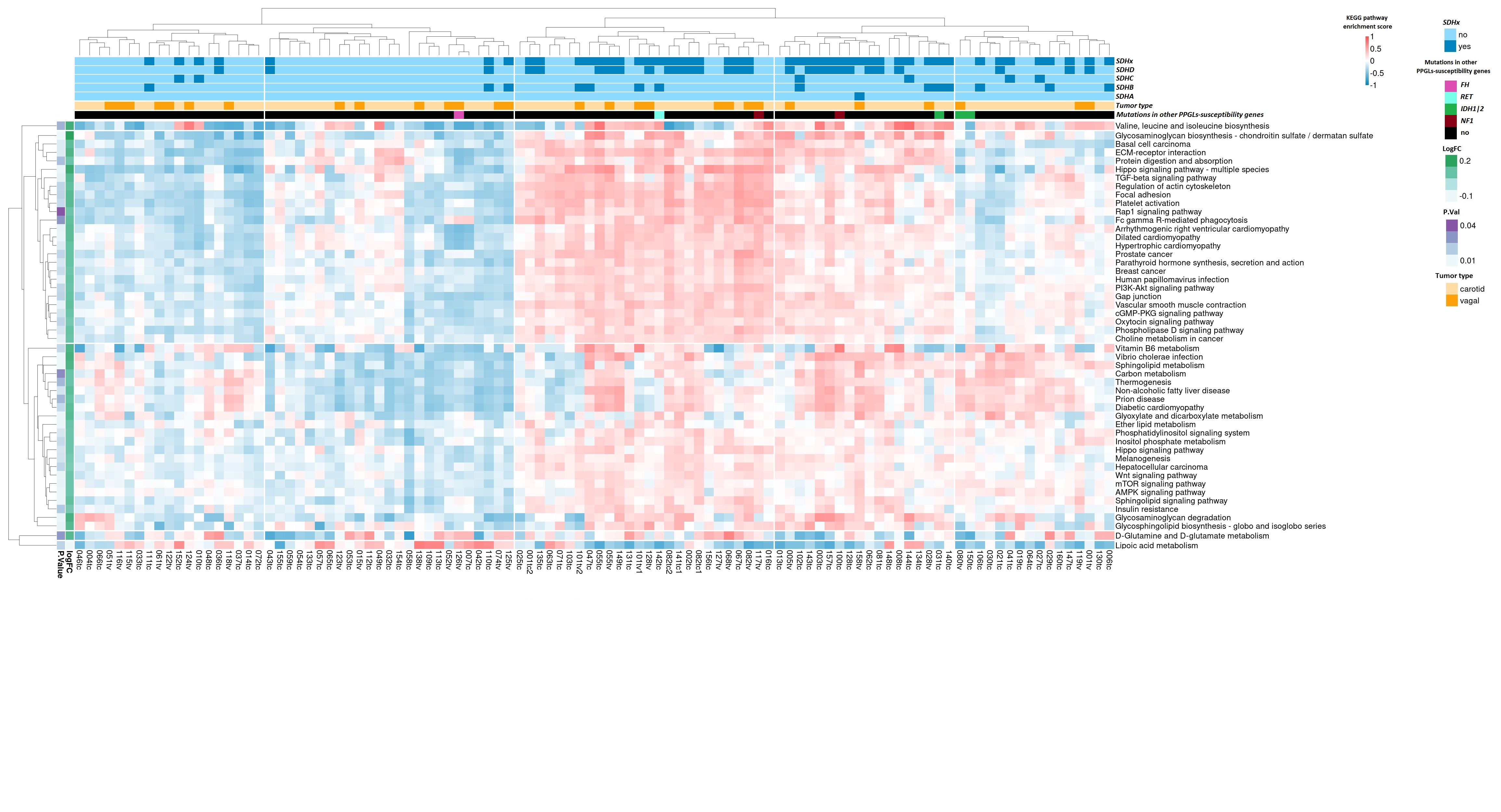


Figure S5. Heatmap of KEGG pathway enrichment scores (top-50) for 104 HNPGLs. The *FH*-mutated tumor is marked with green.


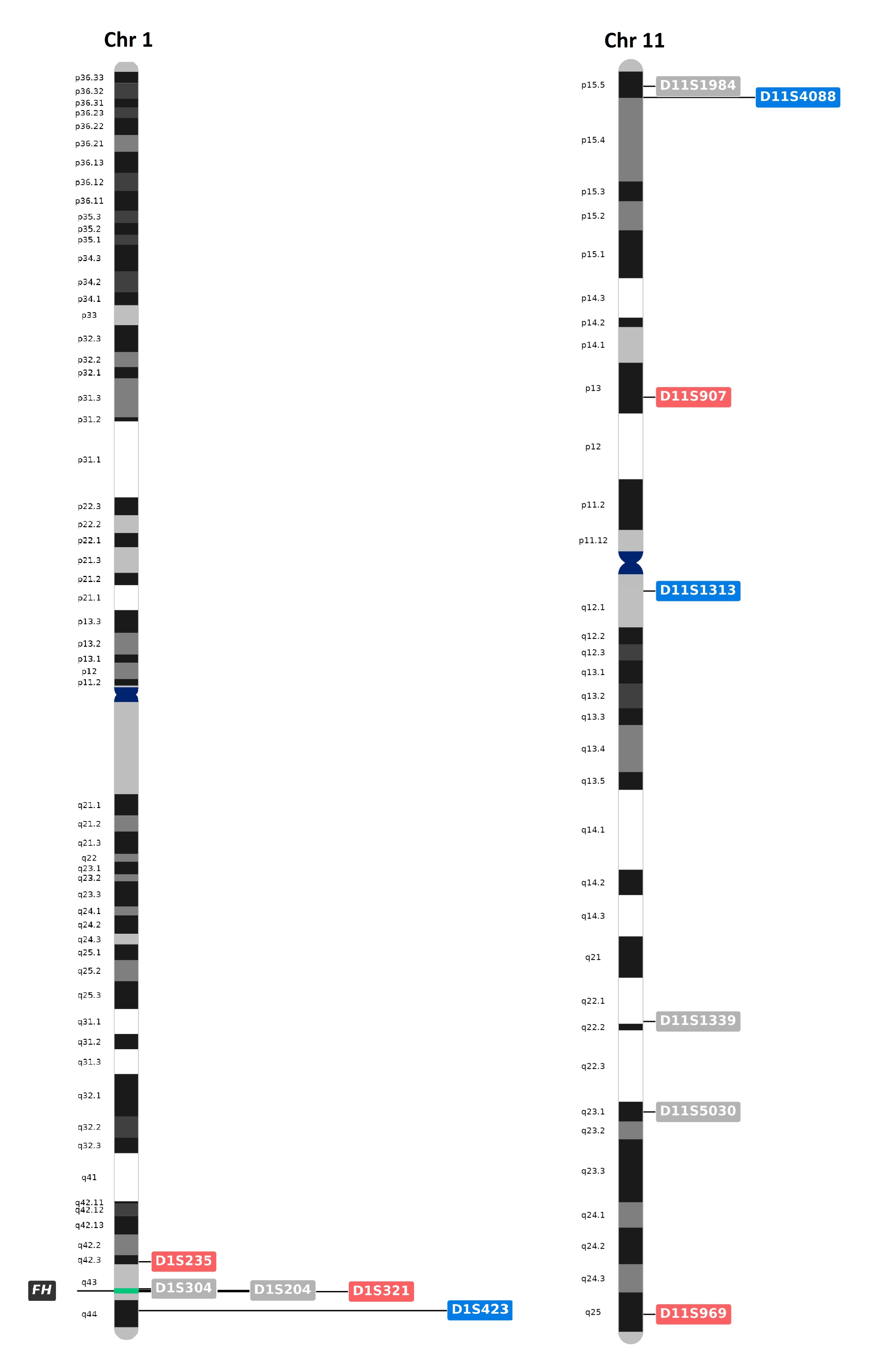


Figure S6. Loss of heterozygosity at the *FH* locus and individual regions across chromosome 11 in *FH*-mutated VPGL. Microsatellite markers show loss (red) or retention (blue) of heterozygosity near the *FH* locus (chromosome 1) and across chromosome 11. Homozygous (non-informative) microsatellite repeats are shown in gray.
